# How to Get Cost-Effectiveness Analysis Right? The Case of Vaccine Economics in Latin America

**DOI:** 10.1016/j.jval.2016.04.014

**Published:** 2016-12

**Authors:** Amanda Glassman, Oscar Cañón, Rachel Silverman

**Affiliations:** 1 Center for Global Development, Washington, DC, USA; 2 Ministry of Health, Bogotá, Colombia

**Keywords:** cost-effectiveness, developing countries, Latin America, vaccination

## Abstract

**Background:**

In middle-income countries, vaccines against pneumococcal disease, rotavirus, and human papilloma virus are in general more costly, not necessarily cost saving, and less consistently cost-effective than earlier generation vaccines against measles, diphtheria, tetanus, and pertussis. Budget impact is also substantial; public spending on vaccines in countries adopting new vaccines is, on average, double the amount of countries that have not adopted. Policymakers must weigh the costs and benefits of the adoption decision carefully, given the low coverage of other kinds of cost-effective health and nonhealth interventions in these same settings and relatively flat overall public spending on health as a share of gross domestic product (GDP) over time.

**Objective:**

This paper considers lessons learned from recent vaccine cost-effectiveness analyses and subsequent adoption decisions in Latin America a, largely under the auspices of the Pro Vac Initiative.

**Results:**

The paper illustrates how small methodological choices and seemingly minor technical limitations of cost-effectiveness models can have major implications for the studies’ conclusions, potentially influencing countries’ subsequent vaccine adoption decisions.

**Methods:**

We evaluate the ProVac models and technical outputs against the standards and framework set out by the International Decision Support Initiative Reference Case for economic evaluation and consider the practical effects of deviations from those standards.

**Conclusions:**

Lessons learned are discussed, including issues of appropriate comparators, GDP-based thresholds, and use of average versus incremental cost-effectiveness ratios as a convention are assessed. The article ends with recommendations for the future.

## Key points

• Cost-effectiveness analyses to inform new vaccine introduction in low- and middle-income countries is increasingly seen as critical to the maximizing the efficiency and impact of vaccination programs.
• This paper considers technical lessons learned from recent vaccine cost-effectiveness analyses and subsequent adoption decisions in Latin America, largely under the auspices of the ProVac initiative. The program has made enormous strides in defining models, sharing expertise with policymakers and promoting use of evidence in vaccine adoption decision using evidence.
• The paper also illustrates how small methodological choices and seemingly minor technical limitations of cost-effectiveness models can have major implications for studies’ conclusions, potentially influencing countries’ subsequent vaccine adoption decisions.

## Introduction

Middle-income countries face special pressures and circumstances when considering adoption of second-wave vaccines, specifically those against rotavirus, human papilloma virus, and the 7, 10, and 13 serotypes of pneumococcal disease (PCV7, PCV10 and PCV 13). These new vaccines are in general more costly, not necessarily cost saving, and less consistently cost-effective in different settings than earlier vaccines against measles, diphtheria, tetanus and pertussis. Budget impact can also be substantial; public spending on vaccines in countries adopting new vaccines is – on average – double the amount of countries that have not adopted. Policymakers must weigh the costs and benefits of the adoption decision carefully, given the low coverage of other kinds of cost-effective health and non-health interventions in these same settings and relatively modest growth in overall public spending on health over time.

This paper considers the technical lessons learned from the past decade of vaccine evaluation and adoption decisions in Latin America, primarily conducted with support from the ProVac initiative. ProVac was launched in 2004 as the first large-scale, systematic effort to support governments in conducting their own economic evaluation of vaccines. A decade later, ProVac is the source of the majority of such evaluations in Latin America and the Caribbean, having achieved notable operational and technical successes while applying a novel approach to an issue of tremendous importance. Yet the Latin American experience over the past decade also shows how seemingly small methodological choices can deeply affect the outcome and utility of economic evaluation, leading to risk of sub-optimal resource allocation decisions. These technical lessons have important implications for the future evolution of the field of economic evaluation and other efforts to support development of more evidence-based priority-setting mechanisms in low- and middle-income countries. The experience also illustrates remaining political barriers to evidence-based decision-making, which are further elucidated in a complementary working paper.

This paper considers the technical lessons learned after ten years of ProVac support for vaccine evaluation in Latin America. The remainder proceeds as follows. Section two discusses the special challenge of second-wave vaccine introduction in middle-income countries; section three then describes how the design of the ProVac initiative addresses gaps in country capacity for evidence-based decision-making around vaccine adoption. With reference to the framework and standards established in the Gates Reference Case for Economic Evaluation [1], the paper next considers how ProVac models rate against established technical standards and key technical lessons learned from the Latin American experience (section four). Finally, the paper concludes with a discussion of how the ProVac model relates to the broader ecosystem of priority-setting in low- and middle-income countries (section five).

## To Adopt or Not to Adopt? The Special Challenge of Second-Wave Vaccine Introduction in Middle Income Countries

The history of mass vaccination has progressed in two major waves. The first wave, taking place in the 1960s and 70s, saw the introduction of several inoculations against major childhood killers, most notably measles, mumps, and rubella (MMR); oral polio vaccine (OPV); and diphtheria, pertussis, and tetanus (DPT). These vaccines were uniformly low-cost, with a price tag of under $1 per dose everywhere in the world; they were also cost-saving in the very short-term, as averted infections also averted the costs associated with treatment and hospitalization. And of course, the averted disease burden represented a major humanitarian achievement, saving many children from death before their fifth birthdays.

The second wave, covering the period between the 2000 and the present day, has seen the introduction of new vaccines against rotavirus, human papilloma virus, and the 7, 10, and 13 serotypes of pneumococcal disease (PCV7, PCV10 and PCV 13). These vaccines are a major medical breakthrough against major causes of death and morbidity. Yet compared to first-wave vaccines, they are in general more costly, not necessarily cost-saving, and less consistently cost-effective. A fourth vaccine introduced during this period, against Haemophilus influenza type B (HiB), falls into an intermediate zone, found to be cost-saving in some settings but not others.

As first-wave vaccinations saved lives and money almost immediately at a very low cost, public payers were eager to subsidize their introduction so long as they had sufficient budgetary space to do so. With the health and fiscal benefits so obviously exceeding the costs, the vaccine introduction decision was relatively straightforward and uncontroversial.

In contrast, the decision to introduce costlier second-wave vaccines can be more complicated, requiring careful attention to affordability and cost-effectiveness compared to other health priorities that compete for scarce public funds. New vaccines must compete for resources against other interventions to target the same disease, as well as against completely unrelated interventions to address other diseases. For example, the rotavirus vaccine can be compared to other health sector interventions to prevent or control diarrheal diseases such as oral rehydration therapy, hygiene education, and breastfeeding promotion; it can also be compared to health sector interventions to prevent or treat malaria, HIV, and tuberculosis. Cost-effectiveness analysis is needed to weigh the costs and benefits of second-wave vaccine introduction and inform an evidence-based decision on adoption.

**Fig. 1:**
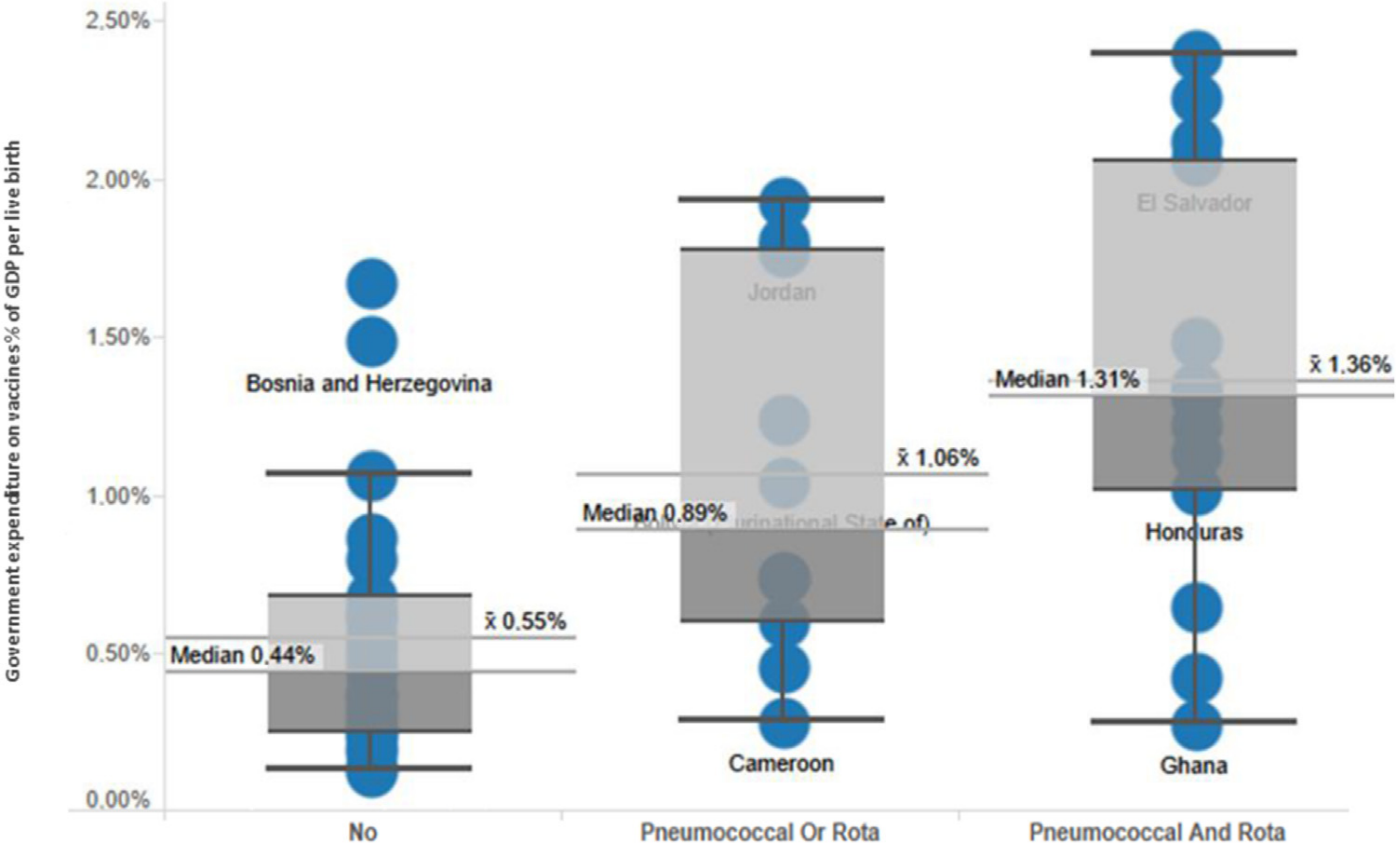
Comparison of government spending on vaccines (2012) sorted by introduced vaccines. GDP, gross domestic product.

The potential budget impact of second-wave vaccines is substantial. As in higher-income countries [2], vaccination budgets are far higher in middle-income countries that have introduced second-wave vaccines when compared to their peers that have not yet done so. Figure 1 compares vaccine expenditure in middle-income countries by introduction status of the pneumococcal and rotavirus vaccines. Public expenditure on vaccines (mean or median) as a share of GDP is more than double (per live birth) when compared to countries that have not introduced the vaccines.

As is self-evident, the price of second-wave vaccines is an important determinant of their overall cost-effectiveness and affordability, making effective price negotiation of paramount importance. Low-income countries (and some transitioning lower-middle income countries) have benefitted from Gavi efforts to pool demand and lead negotiations, enabling lower prices. Most high-income countries, on the other hand, rely on strong domestic institutions and capacity to evaluate the costs and benefits of second-wave vaccines and thereby negotiate moderate prices with industry. Within the current global order, however, many middle-income countries – home to most of the world’s poor and ill, and including most countries in Latin America – are stuck on the uncomfortable middle ground, with some ability to pay but with limited technical and institutional capacity to conduct economic evaluation, assess budget impact, and negotiate effectively on price. The presence of the PAHO’s Revolving Fund has to some extent centralized the issue of price negotiation in Latin America and the Caribbean away from country governments, but not all countries in the region participate, or participate consistently, in the Fund procurement mechanism. Further, outside the PAHO region in non-Gavi countries, price negotiation remains a major challenge.

The challenge facing middle-income countries is compounded by the structure of the market for second-wave vaccines. Although most new vaccines have remained on-patent during the period of this research, almost all products face competition from a comparator vaccine produced and marketed by a second company. For example, the pneumococcal vaccine has been produced by Pfizer as Prevenar© (7 [now retired from market] and 13 valent) and by GSK as Synflorix©; the rotavirus vaccine is produced as RotaTeq© by MSD Sanofi and as Rotarix© by GSK; and the HPV vaccine is produced as Gardasil© by MSD Sanofi and as Cervarix© by GSK. While each company and product has its own pricing strategy in each country (and for each payer agency), in many cases the products compete head-to-head for public sector market share. This market structure has resulted in highly variable vaccine prices, the result of intense negotiations between public purchasers and industry.

**Fig. 2:**
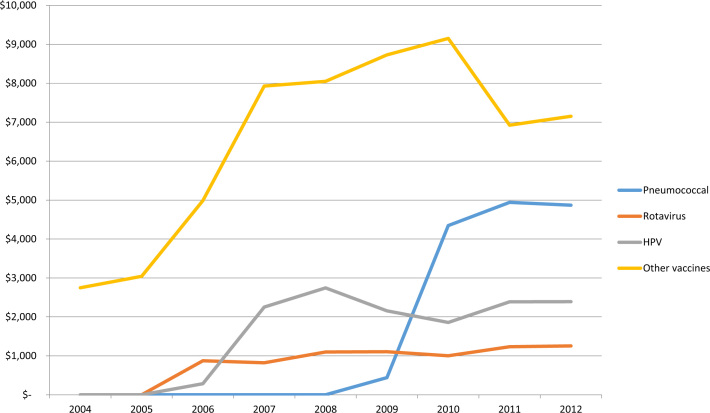
Trends in revenues from vaccines for the three largest firms, 2004–2012 (millions of constant 2013 US dollars). HPV, human papilloma virus.

The vaccine market in middle-income countries is a large and important one for both national and international manufacturers. Globally, vaccine sales amount to $24 billion each year for the three companies that concentrate 70% of the market [3]. About half of these sales are made up by the vaccines against pneumococcal disease, rotavirus and human papilloma virus (HPV) – and that market has grown substantially (Figure 2). In 2011, four of the top fifteen products sold by GSK were vaccines, and vaccines represented 19% of their total sales in 2010. Pfizer’s PCV 13 and 7 were their second most profitable products, generating $969 million worldwide and representing 7% of total revenues.

Availability of multiple products that target the same disease alongside a highly concentrated and profitable market combine to generate marketing pressure of various kinds on public payers in middle-income countries. These decision-makers can be ill-equipped to analyze alternatives and make informed decisions, within a context where overall public spending on health as a share of GDP has remained more or less flat over the past decade. The existence of these pressures on decision-makers, within the context of a relatively stable budget constraint, enhances the relevance and salience of cost-effectiveness analyses in these settings.

## Decision Support for Latin American Policy-Makers: Overview of the ProVac Program

In 2004, in this context of mounting pressure on Latin American policymakers, PAHO’s governing body issued a formal request for technical support for the economic evaluation of second-wave vaccines (Directing Council Resolution CD47.R10). In response, with funding from the Bill & Melinda Gates Foundation, PAHO launched the ProVac Initiative with the goal of “strengthening the technical capacity for evidence-based decision-making on new vaccine introduction.”

**Fig. 3:**
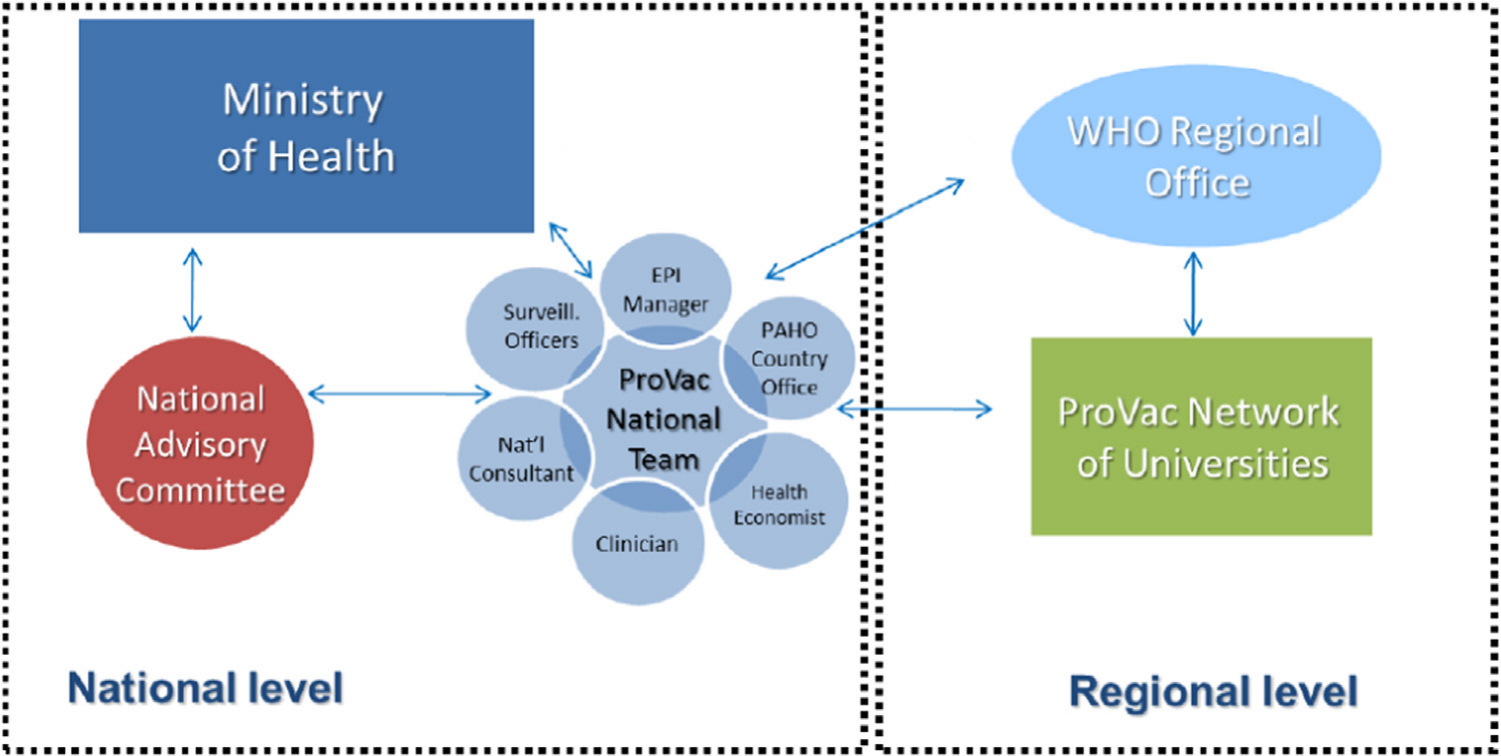
ProVac operating model. EPI, Expanded Program on Immunization; WHO, World Health Organization. *Source*. PAHO ProVac (2013).

ProVac defines its activities as supporting “a clearly defined country-led process for evidence-based analysis that increases ownership and trust in the results by national authorities.” Support is triggered by an official request from the Ministry of Health (MoH) of member countries. Under the ProVac model, PAHO then supports the formal creation of a multi-disciplinary team led by the MoH (Figure 3), begins a training program, and helps the team craft a timeline and workplan for gathering data and conducting the economic analysis, using standardized proprietary tools and models developed specifically for the ProVac initiative. The process ends with a presentation of results to the national authorities, with the goal of informing the decision-making process for vaccine adoption. In 2010, ProVac also established a network of Regional Centers of Excellence (CoE) to help gather regional evidence and develop methodological guidelines and tools [4]. By the close of 2013, ProVac had supported 24 economic analyses in 16 LAC countries [5].

As a result of ProVac support, the vaccine adoption and funding decision, informed by assessment and deliberation, is expected to be “better” – understood for purposes of this study as most health-enhancing at least cost – than a counterfactual where adoption decisions are taken based on inertia, marketing, interest group pressures or regional-level decisions and result in second- or third-best uses of scarce public monies for health.* The goal of the program is therefore not to increase the number of countries opting for second-wave vaccine adoption, but to improve the decision-making process and result in favor of population health and more efficient use of public resources [4].

As ProVac was implemented, the model was expanded to cover low- and middle-income countries in other parts of the world through a supplementary Bill & Melinda Gates Foundation grant for a ProVac International Working Group (ProVac IWG). By the end of 2013, the ProVac IWG had conducted 3 regional workshops and 7 cost-effectiveness analyses.

## Technical Lessons: Cost-Effective Compared to What?

To aid countries in making evidence-based vaccine adoption decisions, the ProVac initiative built cost-effectiveness models and shared technical expertise with country-level decision makers. A full evaluation of the models’ quality against standard criteria defined by defined by Wilkinson et al. (2016) is presented in Appendices 2 and 3 (methods for the evaluation are detailed in Appendix 1) [1]. Broadly speaking, the two ProVac models – CERVIVAC for the HPV vaccine and TRIVAC for the pneumococcal and rotavirus vaccines – have worked well for the initiative, achieving the double objective of being accessible to users that are not expert in cost-effectiveness analysis while still being sufficiently robust to support policy decisions.

However, the core purpose of the program’s technical component, within the broader context of the initiative, was not just to determine the theoretical cost-effectiveness of a vaccine in the abstract but also to inform the adoption and purchasing decision for policymakers in specific settings – policymakers tasked not just with a binary decision to adopt or not adopt, but with a decision between comparator vaccines plus other screening and treatment interventions that could be implemented either individually or in combination. Thus it is important to consider the relevance of the purpose-built tools and technical advice in informing this real-world decision. To do so, we evaluate the ProVac models and technical outputs against the standards and framework set out by the International Decision Support Initiative Reference Case for Economic Evaluation and consider the practical effect of deviations from those standards [1].

As is common in the field of economic evaluation, minor limitations in the ProVac models and approaches had implications for their ability to address core policy questions. The models’ limitations – and the consequent limitations of the studies that emerged from them – are three fold. First, the output of the first generation of ProVac modelling (Trivac) did not allow for direct comparison of all possible interventions, whether between vaccine candidates or non-vaccine interventions. Second, as is standard in the field, the models were programmed to report cost-effectiveness in relation to the WHO-recommended GDP per capita-based thresholds – thresholds that lack theoretical or empirical basis and may not make sense in specific country contexts as a proxy for the affordability of a vaccine. Finally, the calculation of average cost-effectiveness ratios (ACERs) as the built-in function of the program instead of incremental cost-effectiveness ratios (ICERs) may have led to errors in the interpretation of results.

These issues are not specific or unique to ProVac, but are instead features of cost-effectiveness modelling for vaccines in general, and for health technologies and interventions more broadly. Even in high income settings, for example, the standard is often vaccine versus no intervention only, and many models used to inform the World Health Organization’s Strategic Advisory Group of Experts (SAGE) and the US Centers of Disease Control’s Advisory Committee on Immunisation Practices (ACIP) reflect this same approach. Similarly, use of a GDP per capita threshold to determine cost-effectiveness is nearly ubiquitous in the literature [6]. This analysis of ProVac helps illustrate concretely how a different approach may help maximize the utility of these excellent technical models for real-world, evidence-based decision making.

### Omission of All Possible Comparators May Lead to Sub-Optimal Decisions

As defined by Wilkinson et al. (2016), one of eleven essential principles for economic evaluation is that “the comparator(s) against which costs and effects are measured should accurately reflect the decision problem” facing policymakers. That is, the relevant comparator(s) should reflect the plausible range of choices – thus, when there are multiple competing products, models are most useful when they allow for their direct comparison between those competing products, not just a binary choice of ‘do nothing’ versus ‘adoption of each specific product’ [1].

The models from ProVac’s first decade do not easily facilitate direct comparisons between all possible and relevant comparator interventions. Currently, the TRIVAC model allows for a one-way comparison between two possible scenarios: adoption of a single vaccine, or adoption of no vaccine. The output of the CERVIVAC model includes two different comparisons: (1) adoption of a single vaccine versus adoption of no vaccine; and (2) adoption of a single vaccine versus various screening methods. However, neither model is equipped to tell countries which vaccine to adopt when there are multiple competing products for the same disease, nor does the TRIVAC model report whether there are better non-vaccine alternatives to reduce the burden of those same diseases. Where there are two products available and subject to review by decision makers, PAHO recommends running the model once for each product. However, this can lead to errors in the interpretation of results (see the next section). It is also important to note that there may be cases where the differences in effectiveness between the two vaccine candidates are too subtle or insignificant from a statistical perspective to make any difference, in which case it would be appropriate to carry out a comparative cost minimisation analysis of products.

The ability to compare vaccination to non-vaccination interventions that address the same diseases is also a technical challenge, particularly in contrast to comparing two similar vaccine products. However, without this capacity, it is not possible to assure the best use of scarce health funding. The need to include all or most relevant technologies and interventions is a constant theme across different guides and methodological recommendations in the field and the literature. Drummond et al (2008) notes: “Because potential inefficiencies exist in all forms of health-care, all health technologies should be potential candidates for HTA. Otherwise, decision making concerning the use of resources is likely to be distorted” [7]. ISPOR best practice guidelines also signal the importance of a broader approach to the decision problem even if this kind of analysis is later constrained by lack of data: “The availability of data may constrain model development, but the initial discussion of the problem should range broadly and encompass features of the disease and its outcomes for which data may be poor or unavailable. It is important to have a complete picture of the problem, regardless of data availability” [8]. WHO echoes these messages: “WHO guidelines on generalized CEA propose the application of CEA to a wide range of interventions to provide general information on the relative costs and health benefits of different interventions in the absence of various highly local decision constraints” [9].

Where it is not possible to provide all relevant information to the decision-maker, the highly restricted choice of comparators should be highlighted as one of the limitations of the CEA. It should also be explicitly noted – with reference to the literature, where relevant – that other interventions may indeed provide greater value for money. For example, some studies have noted that increased breastfeeding may be more cost-effective than vaccination to reduce the burden of pneumonia. Indeed, this limitation has previously been raised by others who have analyzed the model: “the comparator for the vaccine intervention is do-nothing, which may make vaccination look more cost-effective” [10]. ProVac aims to develop a more universal tool that will evaluate multiple vaccines and eventually any relevant comparator interventions called UNIVAC [11].

In the case of CERVIVAC, for example, the model could simultaneously calculate cost-effectiveness for the following comparators: 1) no vaccination nor screening; 2) only screening; 3) only vaccination; 4) vaccination and screening – and it should offer all four scenarios using each of the two available vaccines. Currently, however, the model addresses just two questions: 1) does the HPV vaccine represent good investment value in my country? And 2) does a new or strengthened screening strategy represent better investment value than current screening in my country? Although the current ProVac questions are responsive to WHO guidelines, many countries would benefit from a model that allowed for comparisons between different mutually exclusive alternatives and between different combinations of non-mutually exclusive interventions. Some non-ProVac studies have taken this approach [12].

### Static GDP per Capita Thresholds Are Not Sufficiently Informative for Decision-Making

ProVac models report cost-effectiveness relative to WHO-recommended static thresholds calculated as a multiplier of countries’ GDP per capita. According to the WHO recommendations, an intervention is considered ‘highly cost-effective’ if the cost per disability-adjusted life year (DALY) averted is under the country’s GDP per capita; ‘cost-effective’ if the cost per DALY is between one and three times the GDP per capita; and ‘not cost-effective’ if the cost per DALY exceed three times the country’s GDP per capita [13]. The models use the WHO-recommended thresholds as their default values.

Use of static cost-effectiveness thresholds based on GDP per capita, though standard in the field, is increasingly understood as a problematic practice that obscures the true opportunity costs of various interventions. The first problem is the use of thresholds in the first place: the binary framing of interventions as either cost-effective or not does not provide sufficient information about whether adopting that intervention is the best possible use of scarce public funds. As Marseille et al. (2015) [14] note, “cost-effectiveness analysis is useful only in the context of the choices available in a particular setting and context – e.g. the budget and technical capacity of a national malaria program or Ministry of Health.” To the contrary, a misapplied threshold approach –where the threshold does not reflect the real resource constraints of the overall system—can fail to contextualize the cost-effectiveness of any given intervention vis-à-vis the cost-effectiveness of alternative health-improving interventions – thus, it does not equip policymakers to prioritize among multiple interventions deemed ‘cost-effective’ by the static threshold when adoption of all such interventions is beyond the country’s budget constraint [6].

The second problem is that the actual choice of threshold (three or fewer times the GDP per capita) lacks theoretical and empirical justification, and thus may not represent true ‘value’ or affordability for a given country [15]. To make this point, Marseille et al. (2015) [13] propose a thought experiment where an intervention is found to save one life-year for each GDP per capita spent – e.g., the intervention is considered highly cost-effective by the WHO standard. Now imagine that all individuals are eligible for that intervention each year. In a typical low or middle income country, simply extending that single intervention to the population would cost the government over 30 times its total budget for health; more than five times its total annual revenue; and the country’s total domestic production. An intervention deemed ‘cost-effective’ by this arbitrary threshold thus clearly would not be affordable in practice. More appropriate cost effectiveness thresholds, e.g. those estimated through analysis of the opportunity cost of health foregone, can thus fall well below the GDP per capita-based thresholds traditionally suggested by the WHO [16].

Instead of merely categorizing interventions as cost-effective or not via a static standard, economic evaluation tools and international decision support will be most helpful if they allow countries to evaluate the cost-effectiveness and affordability of interventions vis-à-vis flexible and locally-appropriate thresholds. Here, a locally appropriate threshold is defined as one that reflect budget availability, social values, and willingness to pay within a specific setting.

In addition, the ability of countries to modify their own thresholds for cost-effectiveness is of particular importance given the dynamics of the vaccine market. The vaccine market is highly reliant on bilateral price negotiations, and better cost-effectiveness models could provide countries with greater leverage in procuring vaccines at a lower price point. For example, countries can benefit from knowing the price at which a given vaccine would become cost-saving, allowing them to use that price as a reference point for their negotiations with manufacturers, as has been done in Thailand [17]. This will be most useful in countries not using the PAHO Revolving Fund or other pooling mechanisms for their purchases.

### Utilization of Average versus Incremental Cost-Effectiveness Ratios Can Be Misleading

To determine whether introduction of a given vaccine would be cost-effective in a given setting, the ProVac models calculate what is known as the average cost-effectiveness ratio, or ACER. In essence, this ratio compares the total cost of an intervention to its total value in terms of life years saved, thus derive the ‘average’ cost-effectiveness.

In situations where there are only two possible options – to either do nothing or to adopt a specific new technology – the ACER is a perfectly adequate tool for determining whether adoption will be cost-effective. The problem arises, however, when there are multiple comparator products or interventions, as discussed previously in this section. In such situations, it becomes necessary to consider whether the more expensive product is cost-effective versus the less expensive product(s) – that is, whether the incremental effectiveness of the more expensive product (defined as additional DALYs averted) outweighs its incremental cost. This ratio – the incremental cost-effectiveness ratio, or ICER – is typically considered the gold standard for economic evaluation to inform decision making.

**Fig. 4:**
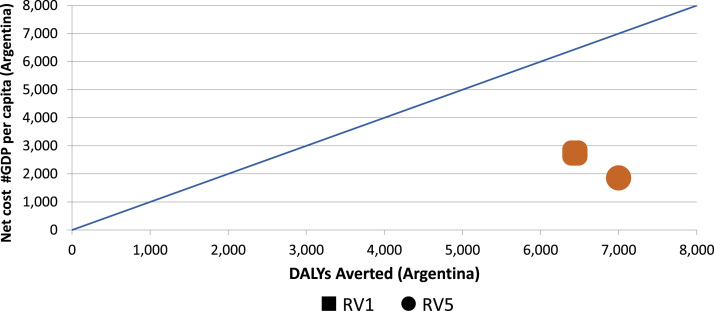
“Dominant” rotavirus vaccine in Argentina identified via ProVac economic evaluations. DALY, disability-adjusted life-year; GDP, gross domestic product; RV1 and RV5, rotavirus vaccines.

To illustrate how ACERs may be misleading in isolation, and how ICERs can be substantially more illuminating, consider the following scenarios. Figure 4 shows the findings of ProVac economic evaluations of rotavirus vaccines in Argentina; the RV 1 vaccine is represented by the square and the RV 5 vaccine by the circle, while the blue line represents the WHO-recommend threshold for ‘highly cost-effective’ interventions. Using ACERs, both vaccines were identified as highly cost-effective when compared to no vaccine vis-à-vis the WHO standard, and the ACER for RV5 would slightly outperform the ACER for RV1. Yet that comparison obscures an essential point: RV5 actually dominates RV1 in Argentina, meaning it is both less expensive *and* more effective. Thus the incremental value of choosing RV1 instead of RV5 would be negative – that is, far from a cost-effective intervention, the ICER would show that RV1 actually subtracts health value for every additional value spent. That determination of its relative value and cost-effectiveness cannot be made by looking at ACERs alone.

Next consider Paraguay, where the published evaluation considered both potential pneumococcal conjugate vaccines (e.g. PCV10 and PCV13). The findings of the evaluation are presented in Table 1. Using the ACER, both the PCV10 and PCV13 vaccines were found to be cost-effective when compared to no vaccination from the social perspective, and PCV10 was found to be highly cost-effective. Once again, however, the ACERs provided a misleading picture of the true vaccine value. A secondary analysis shows that the more expensive vaccine – PCV13 – had an ICER of $15,696, or 6.2 times GDP per capita, far outside even the most generous thresholds for cost-effectiveness [18].

Despite reporting the ICER in a table, the text of the published Paraguay study does not report that the PCV13 is cost-ineffective. In contrast, its analysis focuses exclusively on the ACER, concluding that “PCV10 and PCV13 were both shown to be cost-effective when compared to no vaccination in Paraguay.” The study does not mention or reflect on the fact that the ICER for PCV13 is larger than the 3 GDP per capita that the study adopts as its maximum threshold for cost-effectiveness. (Using evidence from an earlier version of the study Paraguay ultimately adopted PCV10, the more cost-effective vaccine, in March 2012) [18]. Better practice may be to report ACER and ICER together.

In some cases, the ACER for a vaccine might make it appear less cost-effective than a comparator – but because the ICER is actually below the locally appropriate cost-effectiveness threshold in a country, the more expensive vaccine could still be the correct choice for adoption (budget permitting). Under the following hypothetical example (Table 2), Comparator 1 would cost 50,000 times GDP per capita and avert 100,000 DALYs, for an ACER of .5. Comparator 2 would cost 60,000 times the GDP per capita and avert 115,000 DALYs, for an ACER of .52. But the cost of each incremental DALY averted would be just .67 times the GDP per capita – and that ICER would still meet the threshold for a country using the WHO-recommended standard. Thus in almost all scenarios with at least two comparator products, the ICER is the only relevant metric for selecting the optimal product for adoption.

## Conclusions

This paper has noted important lessons derived from the past decade of vaccine evaluation and adoption decisions in Latin America, focusing on those conducted using the technical tools and support offered through the ProVac program. To be most useful, the cost-effectiveness models need to directly address the core decision points faced by countries: not just whether a given intervention would, on average, have benefits that outweigh costs, but also whether that intervention is the best possible use of scarce health resources given a set budget constraint and competing priorities. That implies that in the future, models and technical support should equip countries to consider all possible comparator products -- both vaccine and non-vaccine comparators; to calculate and clearly report the incremental cost-effectiveness ratio (ICER) for each rather than the far less informative average cost-effectiveness ratio (ACER); to critically appraise and select a theoretically sound threshold for cost-effectiveness that reflects social preferences and local context; to estimate the overall budget impact; and to use the results of such analysis to gain greater leverage in price negotiations.

## Figures and Tables

**Table 1:** Parameters for the economic evaluation of RV1 and RV5 in Guatemala

**Parameter**	**RV1**	**RV5**
Price per dose (US $)	7.00	21.79
No. of doses	2	3
Price for all doses (US $)	14.00	65.37
Efficacy against diarrhea of any severity	70% (95% CI 46–84)	74% ( 95% CI 67–80)
Efficacy against severe rotavirus-related diarrhea	85% (95% CI 72–94)	98% (95% CI 88–100)
Efficacy against hospitalization	85% (95% CI 70–94)	95% (95% CI 91–97)
Security (intussusception)	No increase in incidence up to 31 d postvaccination	No increase in incidence up to 42 d postvaccination

CI, confidence interval; RV1 and RV5, rotavirus vaccines.*Source.* Ministerio de Salud Pública y Asistencia Social (2012).

**Table 2:** Hypothetical example when ACERs can be misleading

**Comparator**	**Net cost to government (# GDP per capita)**	**Net DALYs averted**	**ACER (# GDP per capita)**	**ICER (# GDP per capita)**
Comparator 1	50,000	100,000	0.5	0.5
Comparator 2	60,000	115,000	0.52	0.67

*Source: Authors*.

## Footnotes

* Other criteria might also be considered such as equity, financial protection, and ethical considerations; however, the purpose of the ProVac Initiative is to bring evidence on cost-effectiveness vis a vis a health goal to the decision-making process.
