# ERRATUM

**DOI:** 10.1016/j.jval.2017.01.010

**Published:** 2017-03

In: Glassman A, Cañón O, Silverman R. How to Get Cost-Effectiveness Analysis Right? The Case of Vaccine Economics in Latin America. Value Health 2016;19(8):913-20.

In the December 2016 issue of *Value in Health* (Volume 19, Issue 8), Affiliation 2 and Table 1 in the article, “How to Get Cost-Effectiveness Analysis Right? The Case of Vaccine Economics in Latin America,” have been corrected. The previous version contained an unrelated table from an earlier draft.

**Table 1:** Results of the Economic Evaluation of PCV10 and PCV13 in Paraguay, Societal Perspective

	Net Cost (USD Millions)	Net Cost (# GDP per Capita)	Net DALYs Averted	ACER (# GDP per Capita)	ICER (# GDP per Capita)
PCV10	23.68	9,410	12,328	**.76**	**.76**
PCV13	51.58	20,499	14,106	**1.45**	**6.2**

Source: Kieninger et al. (2015). Conversions to GDP per capita scale calculated by authors.

The correct affiliation for Oscar Cañón is ^2^Universidad Jorge Tadeo Lozano de Bogotá, Colombia.

