# Supplementary material for: How to Get Cost-Effectiveness Analysis Right? The Case of Vaccine Economics in Latin America
Source: Value Health. 2016 Dec;19(8):913–20. doi: 10.1016/j.jval.2016.04.014 (PMC5193155; doi:10.1016/j.jval.2016.04.014)
Supplement: Supplementary file 1 — Supplementary material [file mmc1.docx]

**Appendix 1: Methods for Assessment of ProVac Models**

Based on a review of the literature on assessment of economic evaluation models [1] [2] [3] [4] [5], three high-level evaluation criteria were identified under which sub-criteria were developed and assessed: methodology, design, and usability. Under methodology, the team assessed how well the model met its stated objectives. Under design, the team looked at how the decisions made in structuring the model including built-in data values, the way in which data is processed and the way in which results are presented helps with its stated objectives. Under usability, the team assessed ease of use and the model’s capacity to facilitate learning and development of skills.

TRIVAC is a model that allows for evaluation of three different types of vaccines, basically constituting three different models with some common elements. The following documents were used to conduct the assessment:

- “TRIVAC 1.8.01.xls” (42,932 KB)
- TRIVAC decision-support model for evaluating the cost-effectiveness of Haemophilus influenzae type b, pneumococcal and rotavirus vaccination. Clark A, Jauregui B, Griffiths U, Janusz CB, Bolaños-Sierra B, Hajjeh R, Andrus JK, Sanderson C. Vaccine. 2013 Jul 2;31 Suppl 3:C19-29. doi: 10.1016/j.vaccine. 2013.05.045. (Clark et al., 2013a)
- Appendix to: TRIVAC decision-support model for evaluating the cost-effectiveness of haeophilus influenzae type b, pneumococcal and rotavirus vaccination. Clark A, Jauregui B, Griffiths U, Janusz CB, Bolaños-Sierra B, Hajjeh R, Andrus JK, Sanderson C. Vaccine. 2013 Jul 2;31 Suppl 3:C19-29. doi: 10.1016/j.vaccine.2013.05.045. (Clark et al., 2013b)
- PAHO, PROVAC. Workshop Booklet: Exercises for Participants. 59 pages.
- PROVAC; PAHO; WHO; AMP; CDC; SABIN; PATH. Workshop Exercises: Booklet for participants. 53 pages.

The following documents were used to assess the CERVIVAC model, which assesses the cost-effectiveness of HPV vaccines among adolescent females and screening strategies among adult women:

- “CERVIVAC_v1.1.11.xls” (38,046 KB)
- “Plan de trabajo_CERVIVAC.xlsx” (21 KB)
- Preguntas de evaluación del modelo CERVIVAC, Material de entrenamiento de país enero 2013. 11 diapositivas.
- Conceptos básicos sobre evaluaciones económicas y el modelo CERVIVAC. ProVac. Presentación 20 diapositivas.
- Fundamentos de la evaluación económica en salud para los profesionales de salud pública. Anushua Sinha, Cara Janusz, Bárbara Jauregui , Elisa Prieto y el equipo técnico de ProVac. 4 paginas.
- Ejercicio 1 del modelo: Familiarización con el Modelo CERVIVAC de ProVac. 7 páginas.
- Ejercicio 2 del modelo: Comprensión de los PARÁMETROS COMUNES a las evaluaciones de la vacunación contra el VPH y del tamizaje del cáncer cervicouterino. 9 páginas.
- Ejercicio 3 del Modelo: Comprensión de los parámetros del PROGRAMA de VACUNACIÓN. 6 páginas.
- Ejercicio 5 del Modelo: Comprendiendo cómo interpretar los RESULTADOS. 3 páginas.
- Ejercicio 6 del Modelo: Comprendiendo cómo usar el análisis de Escenarios. 6 páginas.

**Appendix 2: Assessment of TRIVAC Model**

| 1. Methodology and conceptualization | | | | | | | |
| --- | --- | --- | --- | --- | --- | --- | --- |
| - 1. Purpose and scope of model is explicit and relevant | - - 1. Study question specified | | YES | | Opportunity for Improvement (OFI) | NO | HTA best practices require the formulation of specific research questions as inputs into the development of a model. Currently, neither the Excel model nor the Vaccine journal methods paper published by the team makes the research question explicit. As Halpern et al (1998) recommend: “When beginning to design or evaluate a model, the question that the model is to answer must be explicitly stated ensuring that the reason for developing the model is made clear.” The CERVIVAC model and supporting documentation represents better practice than the TRIVAC materials. However, the modeling team notes that some of the CEA studies based on the model do better on specifying specific policy questions, and that ProVac training sessions include this aspect. |
|  | - - 1. Spectrum of illness defined | | YES | | OFI | NO | The spectrum of illness is not described in separate section, but does appear in the description of the structure of the model in the methods paper. |
|  | - - 1. Time horizon defined | | YES | | OFI | NO | The model evaluates between one and 20 cohorts. For each cohort, costs and deaths are documented for the first five years. For the estimation of DALYS, life years lost and disability, life expectancy is used. |
|  | - - 1. Perspective defined | | YES | | OFI | NO | The model allows for the analysis of two perspectives, government and society. |
|  | - - 1. Comparators defined | | YES | | OFI | NO | The model allows for three different evaluations (Hib, Pneumoccocal, HPV). In each, it is only possible to compare do-nothing against a single vaccine comparator. However, various comparators can be selected.   - Hib   - Comparator 1: No vaccination   - Comparator 2: Three options, of which only one can be selected:     - Vaccinate against Hib     - Vaccinate against Hib + DTP     - Vaccinate against Hib + DTP + HepB - Pneumoccocal   - Comparator 1: No vaccination   - Comparator 2: Three options of which only one can be selected     - Vaccinate against Pneumoccocal with PCV7 Prebnar^® (1st version)^     - Vaccinate against Pneumoccocal with PCV10 Synflorix^®^     - Vaccinate against Pneumoccocal with PCV13 Prebnar^® (2nd version)^ - Rotavirus   - Comparator 1: No vaccination   - Comparator 2: Two options of which only one can be selected:     - RV1 – Rotarix^®^     - RV5 - Rotateq^®^   In its current design, it’s neither possible to directly compare two vaccines, nor to include non-vaccine interventions. |
|  | - - 1. Population, country and region characteristics are specified | | YES | | OFI | NO | Population, country and regional variables can be adjusted in the model. |
| - 1. Connected with experts and stakeholders interested in economic evaluation of vaccines | - - 1. Model developers’ individual and group conflicts of interest are stated. | | YES | | OFI | NO | In the Vaccine methods paper on TRIVAC, conflicts of interest are declared by the authors of the article. However, given that the Excel model can be distributed separately from the article (in fact, the model was used for a number of years prior to the publication of the article in 2013), it would be desirable that all files and documents that make up the model clearly specify the authors and their possible conflicts of interest. In addition, it is unclear whether the model developers are exactly the same as the authors of the Vaccine article. |
| - 1. Model transparency | - - 1. Authors and affiliations are presented. | | YES | | OFI | NO | See criteria 1.2.1: authors are specified within the journal article, but not within the model itself. |
|  | - - 1. Effort has been made to make all or most information related to the model public | | YES | | OFI | NO | The model does not refer to or utilize privately-held information or parameters that have been endogenized or masked in any way. All parameters are clearly established together with the source of information. These data are preloaded into the model and the model is designed for the validation and update of the built-in data by each country team. |
|  | - - 1. Model includes a description accessible to a broad public | | YES | | OFI | NO | Yes, the Excel file includes a tab titled “Description” with a clear description in accessible language, suited for a broad readership. This description is supported by the Vaccine article. |
|  | - - 1. Model includes technical documentation allowing for an expert to replicate. | | YES | | OFI | NO | The Vaccine methods article includes a technical description. In addition, the Excel model program shows formulas and macros without any protection so that it is possible to replicate the model. |
|  | - - 1. Model is available in the public domain allowing for validation by third parties. | | YES | | OFI | NO | The model has provided to the evaluation team, however, it is unclear what kind of license accompanies the model. The model is not available in the public domain. |
| 1. Design | | | | | | | |
| - 1. Type of model and reasons for its selection are specified and discussed. | | | YES | OFI | | NO | The Vaccines methods article reports that TRIVAC is a static cohort model. It is not clear whether and how the model is related to any of the classic CEA models (table of payments, decision trees, Markov models, other discrete state models). Revising the Excel model, TRIVAC seems to employ a table of payments approach. The Vaccine article discusses other options for the design of the model, including a dynamic model. |
| - 1. Model defines and considers all technologies and relevant treatment strategies. | | | YES | OFI | | NO | Hib: Although not explicitly addressed, it is understood that the evaluated option is prophylaxis against infection caused by the Haemophilus influenzae type b in under 5 year old children. However, among the built-in comparators, there are two that are actually not indicated for Hib prophylaxis. These strategies are DTP and HepB vaccination. The model does not offer the option to compare PCV7, PCV10 and PCV13 amongst themselves, only each type of vaccine against a no vaccination scenario. For this reason, the model cannot be said to define and consider all technologies and relevant treatment strategies. Although there have been studies on other types of vaccines against pneumoccocal disease (Chan-Acón et al., 2010), the vaccines included in the model PVC7, PCV10 y PCV13 are the most relevant (World Health Organization, 2012).  Rotavirus: The model does not offer the option to compare RV1 and RV5 against each other, but only against a no vaccination scenario. For this reason, the model cannot be said to define and consider all technologies and relevant treatment strategies. As with Hib, RV1 and RV5 represent all the relevant strategies for prophylaxis via vaccination against rotavirus (Centers for Disease Control and Prevention, n.d.). |
| - 1. Model structure allows for adequate valuation of costs and outcomes | - - 1. ***Estimation of costs*** | | | | | | |
|  | - - - 1. Model presents analysis from a social perspective. | | YES | OFI | | NO | The model is designed to simultaneously evaluate both government and societal perspectives.  In the societal perspective, government costs as well as household costs are considered.  Within government costs: 1) the cost of administering the vaccine, disaggregated by the cost of security boxes, syringes and waste, as well as a marginal cost associated with the inclusion of a new vaccine and the cost of start-up of the new program; 2) the cost of an outpatient visit by condition and type of facility; 3) the cost of hospitalization by condition and type of facility; 4) the cost of complications.  Within household costs:  1) the cost of an outpatient visit by condition and type of facility; 2) the cost of hospitalization by condition and type of facility; 4) the cost of complications. No other direct (transport, etc.) or opportunity costs are considered.  Adverse reactions to vaccines are not considered from either perspective. |
|  | - - 1. ***Estimation of outcomes*** | | | | | | |
|  | - - - 1. Wide range of outcomes and evidence are considered. | | YES | | OFI | NO | DALYs are the principal outcome analyzed in the model. In addition, life years gained, avoidable deaths, avoidable hospitalizations and avoidable cases are also calculated as part of the model. The number of cases of each type of complication, the number of outpatient visits and the number of hospitalizations associated with disease complications are also generated. |
|  | - - - 1. All relevant outcomes for policymakers and stakeholders are considered, and outcomes are directly related to the model’s study question | | YES | | OFI | NO | While a study question is not specified explicitly, it is clear that averted DALY lost and cost per DALY averted is the relevant outcome. Other outcomes that might be relevant for decision-makers might include the number of QALY gained and the cost per QALY gained.  From a public health and safety perspective, it would be useful to include the number of expected adverse reactions, whether frequent or rare, taking into account that TRIVAC is conceptualized as a modeling tool for the evaluation of large-scale vaccination programs, where it is likely to observe even rare adverse events. |
|  | - - - 1. Sources for all outcomes are specified. | | YES | | OFI | NO | Yes, the model specifies the sources of the outcomes that are pre-loaded in the model and that can be updated and validated by country teams. |
| - 1. Results, outputs of model | - - 1. Model estimates uncertainty associated with results. | | YES | | OFI | NO | The model allows for 4 kinds of sensitivity analysis to approximate the level of uncertainty in the estimations. These include: univariate or one-way analysis; scenario analysis; sub-group analysis; and simple probabilistic analysis. |
|  | - - 1. Base case scenario is presented and described. | | YES | | OFI | NO | The base case scenario is presented and described. |
|  | - - 1. Sensitivity analysis can be carried out: univariate, multivariate, by scenarios, by thresholds | | YES | | OFI | NO | See 2.4.1 |
|  | - - 1. Variables that most affect cost are identified. | | YES | | OFI | NO | The Vaccine methods article includes a discussion of the main variables that affect the cost and cost-effectiveness of the intervention. In the Excel model, graphics can be generated that allow for the analysis of the impact of these main variables on cost-effectiveness. However, a standard tornado diagram is not part of the model. |
| - 1. Entry data included in model | - - 1. Sources of information and quality of evidence are specified. | | YES | | OFI | NO | Given that many countries are not able to access better information than the built-in data included in the model, it would be useful to include a quality assessment of each parameter within the model itself. One option is to reference the ProVac-OLIVES database directly within the Excel model, which includes a field for data quality of included sources. However, for a number of ProVac OLIVES sources, the data quality field is empty and this would require some additional development.^[[1]](#footnote-1)^ |
|  | - - 1. Entry parameters are described. | | YES | | OFI | NO | Yes, entry parameters are clearly described and grouped within one of the worksheets of the model, titled “inputs”. |
| - 1. Assumptions embedded in model are described | | | YES | | OFI | NO | The Vaccine journal article does not include a specific sub-heading under which the assumptions are discussed. However, the article does include a brief discussion around some of the model’s assumptions. It would be useful for the model documentation to explicitly list all assumptions. For example, the model assumes that there are no costs or outcomes related to adverse events related to vaccination, or that these are marginal. This assumption should be made explicit. |
| - 1. Possible biases of the model are explored and analyzed. | | | YES | | OFI | NO | The Vaccine article mentions a few limitations of the model, but the biases are not analyzed explicitly, nor how their direction or magnitude could affect results. The word “bias” is not mentioned. |
| - 1. Verification and validation of the model has been carried out | | - - 1. External peer reviewers have assessed the model. | YES | | OFI | NO | In the documents reviewed, there is no reference to a peer review or validation process. ProVac program notes that reviewers of Vaccine article and QUIVER reviewers were given full access to the model. |
| 1. Usability by country teams and capacity to develop knowledge and skills | | | YES | | OFI | NO | The model provides clear instructions for use, data entry, results generation, graphics and results interpretation. Each input and output parameter is clearly signaled and some more complex parameters have a help window attached with a detailed description. There is also a worksheet (back of the envelope) that describes the steps behind the calculations carried out by the model.  Further, the model has an English and a Spanish setting that allows for broader use. However, if a team wished to dive deeper on the calculation approach or the model’s programming, there is not a guide available. |

**Appendix 3: Assessment of CerviVac**

| 1. Methodology and conceptualization | | | | | | |
| --- | --- | --- | --- | --- | --- | --- |
| - 1. Purpose and scope of model is explicit and relevant | - - 1. Study question specified | | YES | OFI | NO | Within the Excel model, two study questions are identified:  *“1. Does the HPV vaccine have a good investment value in my country? 2. Does a new or strengthened screening strategy have a better investment value than current screening in my country?”*  In the presentation materials attached to the model, the following study question are included:  1-Vaccination:   1. “What is the cost-effectiveness of the HPV vaccine for the prevention of cervical cancer?   2-Screening:  a) “What is the cost-effectiveness of change the current strategy to alternate option X (ej. VIA, DNA testing, etc.)?”  b1)”What is the cost-effectiveness of increasing the coverage of the current program?”  b2)”What is the cost-effectiveness of implementing the current program according to protocol?” |
|  | - - 1. Spectrum of illness defined | | YES | OFI | NO | The spectrum of illness is not clearly defined, although it is possible to deduce that the model refers to infection, lesions and neoplasia caused by HPV. |
|  | - - 1. Time horizon defined | | YES | OFI | NO | The time horizon is the life expectancy of a cohort. |
|  | - - 1. Perspective defined | | YES | OFI | NO | The model allows for the analysis of two perspectives, government and society. |
|  | - - 1. Comparators defined | | YES | OFI | NO | The model allows for two kinds of evaluation, evaluation of vaccination against HPV and evaluation of screening strategies. In each category, it is only possible to compare two options at a time.   1. In vaccination, the comparators are:    1. No vaccination    2. Vaccination, although the product is not specified in any of the entry fields 2. In screening, it’s possible to compare two of the following options:    - PAP (Papanicolaou)    - VIA (visual inspection with acetic acid)    - PAP and self-administered DNA testing    - VIA and self-administered DNA testing    - PAP and clinic-based DNA testing    - VIA and clinic-based DNA testing   In the screening evaluation, it is not possible to evaluate against no screening, nor is it possible to directly evaluate the options of “neither vaccinate nor screen”, “only vaccinate”, “only screen” or “vaccinate and screen.” |
|  | - - 1. Population, country and region characteristics are specified | | YES | OFI | NO | Population, country and region variables can be modified in the model. |
| - 1. Connected with experts and stakeholders interested in economic evaluation of vaccines | - - 1. Model developers’ individual and group conflicts of interest are stated. | | YES | OFI | NO | It is not clear which groups or authors participated in the development of the model, nor conflicts of interest. In the window “About Model”, thanks are given to Gupta, Arnett, Bell, Davison and Morrison, but model developers are not specified. |
| - 1. Model transparency | - - 1. Authors and affiliations are presented. | | YES | OFI | NO | See criteria 1.2.1 |
|  | - - 1. Effort has been made to make all or most information related to the model public | | YES | OFI | NO | The model does not refer to or utilize privately-held information or parameters that have been endogenized or masked in any way. All parameters are clearly established together with the source of information. These data are preloaded into the model and the model is designed for the validation and update of the built-in data by each country team. |
|  | - - 1. Model includes a description accessible to a broad public | | YES | OFI | NO | Yes, the Excel file includes a sheet titled “Description” where key features are described in accessible language. |
|  | - - 1. Model includes technical documentation allowing for an expert to replicate. | | YES | OFI | NO | The model is programmed in Excel with unprotected formulas and macros that allow for replication. |
|  | - - 1. Model is available in the public domain allowing for validation by third parties. | | YES | OFI | NO | The model has been provided for the purposes of the evaluation, however, it is unclear what type of license accompanies the model, and the model is not available in the public domain. |
| 1. Design | | | | | | |
| - 1. Type of model and reasons for its selection are specified and discussed. | | | YES | OFI | NO | The model does not specify the type of model; however, in reviewing the Excel model, it seems to be based on a table of payments model. Neither the Excel nor other available documentation discusses the other options for model design. |
| - 1. Model defines and considers all technologies and relevant treatment strategies. | | | YES | OFI | NO | The model does not permit direct comparisons between two vaccine products, nor between vaccination and different screening strategies. Although more alternatives are included in CERVIVAC than TRIVAC, it is not easily possible to consider all relevant comparators in the same analysis. |
| - 1. Model structure allows for adequate valuation of costs and outcomes | - - 1. ***Estimation of costs*** | | | | | |
|  | - - - 1. Model presents analysis from a social perspective. | | YES | OFI | NO | The model is designed to simultaneously evaluate vaccination and screening from both a social and government perspective.  The social perspective includes government costs and household costs.  The following government costs are included: 1) cost for treatment by type of cancer; 2) cost to administer the vaccine, disaggregated by costs of secure boxes, syringes and waste, as well as start-up costs associated with introduction; 3) cost of screening which includes screening methods and settings, cost of colposcopy by setting, and cost of treatment of lesions by setting.  The following household costs are included:  1) the cost of an outpatient visit by condition and type of facility; 2) the cost of hospitalization by condition and type of facility; 4) the cost of complications. No other direct (transport, etc.) or opportunity costs are considered.  Household costs related to vaccination or cancer sequelae are not considered, nor are government/household costs related to adverse reactions to the vaccine. |
|  | - - 1. ***Estimation of outcomes*** | | | | | |
|  | - - - 1. Wide range of outcomes and evidence are considered. | | YES | OFI | NO | The main outcomes analyzed in the model are averted DALY. In addition, the model generates other outcomes such as life years gained, averted deaths, averted hospitalizations and averted cases, as well as averted cases by stage of cancer.    The model includes built-in sources for the estimation of the parameters used to calculate these outcomes. However, the quality of these references and their representativeness of all of the evidence available is unclear, in part because the model has been designed so that the information can be validated and updated by country teams. |
|  | - - - 1. All relevant outcomes for policymakers and stakeholders are considered, and outcomes are directly related to the model’s study question | | YES | OFI | NO | According to the study questions identified by the model, averted DALY and cost per averted DALY are relevant outcomes. Other outcomes may be relevant for decision-makers such as the number of QALY gained and the cost per QALY gained. From a public health and safety perspective, it may also be relevant to include the number of expected adverse events given that CERVIVAC is a model used for the evaluation of at-scale vaccination programs. |
|  | - - - 1. Sources for all outcomes are specified. | | YES | OFI | NO | Yes, the model specifies the sources of the outcome parameters that are built into the model, and that can be updated and validated by country teams. |
| - 1. Results, outputs of model | - - 1. Model estimates uncertainty associated with results. | | YES | OFI | NO | The model allows for univariate and scenario analyses. |
|  | - - 1. Base case scenario is presented and described. | | YES | OFI | NO | The model presents a base case separate from sensitivity analyses. |
|  | - - 1. Sensitivity analysis can be carried out: univariate, multivariate, by scenarios, by thresholds | | YES | OFI | NO | Only univariate and scenario analyses are available. |
|  | - - 1. Variables that most affect cost are identified. | | YES | OFI | NO | The model provides graphics that identify which variables most affect the cost-effectiveness ratio, although standard tornado charts are not provided. |
| - 1. Entry data included in model | - - 1. Sources of information and quality of evidence are specified. | | YES | OFI | NO | Given that many countries are not able to access better information than the built-in data included in the model, it would be useful to include a quality assessment of each parameter within the model itself. One option is to reference the ProVac-OLIVES database directly within the Excel model, which includes a field for data quality of included sources. However, for a number of ProVac OLIVES sources, the data quality field is empty and this would require some additional development. |
|  | - - 1. Entry parameters are described. | | YES | OFI | NO | Yes, entry parameters are clearly described and conveniently grouped under one worksheet titled “*inputs*”. |
| - 1. Assumptions embedded in model are described. | | | YES | OFI | NO | In evaluated documents, assumptions are not made explicit. |
| - 1. Possible biases of the model are explored and analyzed. | | | YES | OFI | NO | In evaluated documents, biases are not made explicit. |
| - 1. Verification and validation of the model has been carried out | | - - 1. External peer reviewers have assessed the model. | YES | OFI | NO | In evaluated documents, there is no reference to a peer reviewer or validation process for the model. |
| 1. Usability by country teams and capacity to develop knowledge and skills | | | YES | OFI | NO | The model clearly describes instructions for use, data entry, results generation, and graphics development. Each input and output criteria have been clearly signaled and more complex parameters include a help window including a more detailed description. There is also a worksheet (back of the envelope) that describes the steps behind the calculations carried out by the model. Further, the model has English and Spanish settings that allows for broader use. However, if a team wished to dive deeper on the calculation approach or the model’s programming, there is not a guide available. |

**Appendix 4: Assessment of Quality of Country Studies**

One way to assess whether and how much capacity was built is to assess the quality of the product produced using the enhanced capacity, in this case the economic evaluation studies supported by the ProVac Initiative. This is merited as the studies are the principal products/outputs produced by the Initiative, and are based – at least in part – on the models developed by the initiative.

This assessment uses the instrument defined in the Bill & Melinda Gates Foundation’s Methods for Economic Evaluation Project (MEEP) run by NICE International to assess the quality of the studies undertaken with ProVac support. To the MEEP set of standards, the authors added: analysis of all authors to understand the networks of research and cooperation generated by the program; analysis of comparators in greater detail; assessment of unit prices used in studies to understand if likely market price is used; analysis of costs included; assessment of and rationale for the cost-effectiveness threshold used.

ProVac-supported studies are contrasted to non-ProVac vaccine cost-effectiveness studies on new vaccines undertaken with other sources of funding, as a crude means to assess a counterfactual situation of studies conducted without support for capacity development. All published and unpublished studies on pneumococcal, rotavirus, HPV and influenza vaccines undertaken in PAHO member countries (except the United States and Canada) were included in the analysis, with no date or language restriction.

This approach to assessing capacity has clear limitations; there is no real counterfactual (what would have happened without ProVac intervention) and there are many other variables influencing the quality of the studies produced that may go beyond ProVac’s own capacity to control, such as available data, skills and experience of government staff participating in study team, among others. It is important to recognize that many published studies are authored by industry and/or experts in the field, while ProVac studies are authored by newly trained professionals, which may reveal the limitations of their limited exposure to the concepts and to the field. Further, the quality of the studies does not reflect their actual use in decision-making.

Published studies were identified using a search strategy on the web pages of the institutions involved in the project, database searches (Pubmed, Embase, Bireme, Google Academic) and snowball searches. Each reference identified was systematized and classified into a bibliographic database using the Mendeley software. Key words^[[2]](#footnote-2)^ and search strategies are described in detail in Annex 3, and additional studies were provided directly by the ProVac Initiative. 42 economic evaluations were identified that met the inclusion criteria (figure A1). The detail of the results obtained by disease, country, and source of support is presented in tables A1 and A2.

Figure A1: Review prism


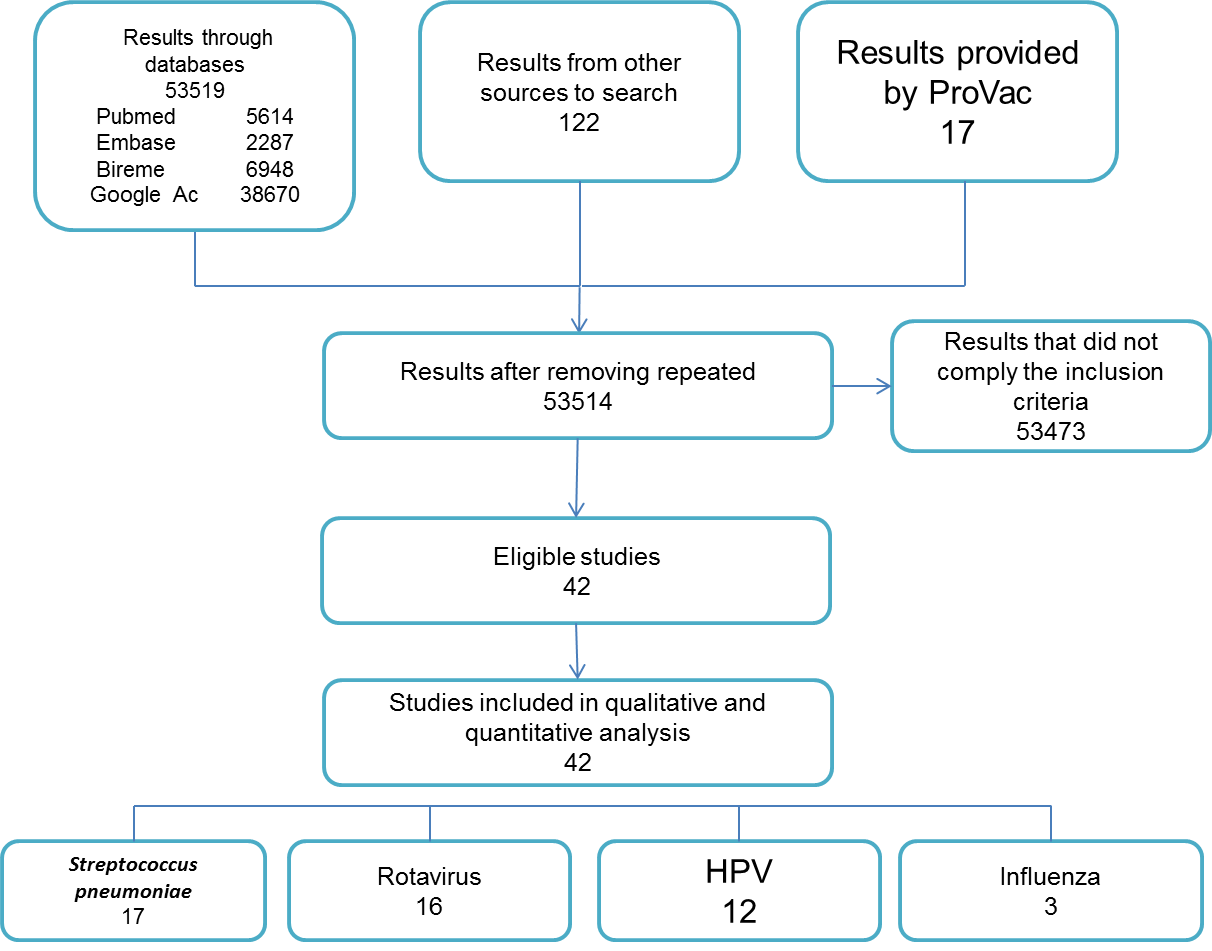


Table A1. Number of studies by disease, ProVac or non-ProVac

|  | Streptococcus pneumoniae | Rotavirus | *Human* papillomavirus | Influenza | Total |
| --- | --- | --- | --- | --- | --- |
| Provac | 8 | 3 | 6 | 0 | 17 |
| Not Provac | 9 | 7 | 6 | 3 | 25 |
| Total | 17 | 10 | 12 | 3 | 42 |

Table A2. Number of studies by country, ordered by total number of studies

|  | Streptococcus pneumoniae | Rotavirus | *Human* papillomavirus | Total | Not ProVac | ProVac | Gross domestic product per capita, current prices ($US 2011) |
| --- | --- | --- | --- | --- | --- | --- | --- |
| BRA | 3 | 3 | 3 | 9 | 9 |  | $ 12,583.64 |
| CHL | 1 | 1 | 0 | 2 | 2 |  | $ 14,551.69 |
| URY | 1 | 0 | 0 | 1 | 1 |  | $ 13,784.56 |
| ARG | 3 | 2 | 0 | 5 | 3 | 2 | $ 10,958.90 |
| VEN | 0 | 1 | 0 | 1 | 1 |  | $ 10,886.05 |
| MEX | 2 | 3 | 3 | 8 | 8 |  | $ 10,033.52 |
| CRI | 1 | 0 | 0 | 1 |  | 1 | $ 8,891.78 |
| PAN | 0 | 1 | 0 | 1 | 1 |  | $ 8,722.22 |
| COL | 3 | 1 | 0 | 4 | 4 |  | $ 7,182.36 |
| PER | 2 | 1 | 0 | 3 | 2 | 1 | $ 5,943.85 |
| DOM | 0 | 1 | 0 | 1 | 1 |  | $ 5,535.34 |
| ECU | 1 | 0 | 1 | 2 |  | 2 | $ 5,324.55 |
| JAM | 0 | 0 | 1 | 1 |  | 1 | $ 5,251.25 |
| ALB | 0 | 1 | 0 | 1 |  | 1 | $ 3,964.68 |
| SLV | 1 | 0 | 0 | 1 |  | 1 | $ 3,696.33 |
| PRY | 0 | 0 | 1 | 1 |  | 1 | $ 3,666.30 |
| GTM | 1 | 1 | 1 | 3 |  | 3 | $ 3,236.13 |
| HND | 0 | 1 | 1 | 2 | 1 | 1 | $ 2,269.51 |
| BOL | 1 | 0 | 1 | 2 |  | 2 | $ 2,269.35 |
| NIC | 1 | 0 | 0 | 1 |  | 1 | $ 1,606.94 |

Searches and analysis were conducted in 2013 up to December of that year. The analysis reflects the studies available at that time. At that time, most ProVac evaluations were unpublished; only one study had been published in a peer-reviewed journal and two studies had been published on a government web page.[6] [7] In the interim, many of the studies have been peer reviewed and released in a special supplement of *Vaccine*. However, the studies reviewed here are the unpublished versions available at the time of analysis.

Most studies use a societal perspective for the evaluation base case (figure A2), whether ProVac or non-ProVac studies, followed by a health system (or government) perspective. All studies used the same discount rate for costs as for benefits (figure A3). ProVac studies sometimes used a higher discount rate than non-ProVac studies. Although the discount rate is always defined in the ProVac models, a portion of ProVac studies did not directly state the discount rate used in the report itself (10% of studies).This is important as deliberation and decisions around vaccine adoption are based on the report not the Excel model.

Figure A2. Number of study perspectives adopted in the base case

Figure A3. Discount rate used in the base case

Figure A4 illustrates the variability in the time horizons used in the studies. Non-report of time horizons was lower in ProVac evaluations. The great variability in time horizons reported in ProVac evaluations is noticeable, in spite of ProVac modelers comments that: “In TRIVAC countries usually follow 10 or 20 cohort s from birth until death. For CERVIVAC (vaccination), they follow 1 cohort from age 9 until death. For CERVIVAC (screening) they follow 1 cohort from age 20 until death. Thus there should only be a maximum of 4 types of horizon, and given the lack of screening evaluations, I would suspect just three.” Economic evaluations of vaccines tend to have more complex time horizons than other types of health products. Various cohorts are usually simulated and followed in terms of cost and out to life expectancy - for the calculation of DALYs. Some of the more complex models include increasing life expectancy for later cohorts. However, the ProVac studies could be clearer on the implications of the time horizon used for policymakers in order to clearly convey the time at which both the costs and benefits described in the ICER are actually realized.

The time horizon should also be assessed jointly with the discount rate. In general, studies showed a time preference for a 3% discount rate for time horizons of more than 80 years. This choice is explained by the fact that 3% is a frequent recommendation in economic evaluation guides that have been developed in countries with low inflation rates, low interest rates and –in general- inter-temporal preferences that are vastly different from those observed empirically and in policy in Latin American countries. It is noticeable that –in spite of the sensitivity of the model to discount rate assumptions- none of the country teams discuss the pertinence of these international recommendations in their own context. In Latin America, however, this may be an underestimate of inter-temporal preferences of policymaker and the population. Discount rates used in other kinds of cost-benefit analyses by ministries of finance come to 10-20%, [8] and it is unclear why a WHO guideline (based on wealthier countries with stable macroeconomic settings) would be indisputably better than a Ministry of Finance recommended rate for cost-benefit studies. Interest rates on borrowing top 25% annually in most countries of the region, which suggests an extremely high valuation of the present versus the future. The discount rate has great importance in these models, as it is the discount rate that most affects ICER results in sensitivity analyses. While 3% is the standard used in the literature, other empirical techniques to determine the discount rate may be merited in Latin America. Scenario analyses –not always included in the available write-ups of country studies and related presentations- were reported to have used varying discount rates by ProVac staff.

Figure A4. Time horizon used in the base case

Figure A5 illustrates that univariate analysis is the only kind of sensitivity analysis undertaken for both groups. More complex probabilistic analyses could be an important step towards making more accurate estimates of the uncertainty associated with the results obtained. This is doubly important given that the univariate analyses show how sensitive the models are to a few variables or a single variable, so it is important to understand how the interaction of all the variables affects results.

Figure A5. Number of studies conducting each type of sensitivity analysis

*Reporting Quality*

In terms of reporting quality, Figure A6 illustrates that most evaluations fail to disclose the funder of the study and to describe the method used to index prices. In general, better reporting practices are observed in non-ProVac evaluations, likely a result of their review and publication in peer-reviewed journals. Only one ProVac evaluation discloses author conflicts of interest, and few provide a date reference for prices used in the evaluation or report an incremental cost-effectiveness ratio (ICER). In four categories, ProVac evaluations were closer to best practices, including equity, time horizon, method used to adjust costs and inclusion of most relevant payers.^[[3]](#footnote-3)^

Of particular value is ProVac’s effort to include all the comparator vaccine products in economic evaluations, something not observed in industry-funded studies where competitor products are not assessed. Unfortunately, most ProVac evaluations that did include more than two comparators (ie, no vaccination plus the two competitor products) report only average cost-effectiveness with respect to no vaccination, and do not report the ICER of the products against each other. Indeed, the only ProVac study reporting an ICER was the study published in a peer-reviewed journal. [9] Among the reporting quality issues reviewed, the non-report of comparative ICER is among the most serious since average cost-effectiveness ratios will not reflect the opportunity cost.^[[4]](#footnote-4)^ And as a result, ProVac studies conclude that both products are cost-effective when compared to no vaccination. However, the calculation of the ICER is necessary to provide accurate policy guidance (see Box 1). Non-calculation of the ICER may be explained by the fact that the TRIVAC and CERVIVAC models do not calculate the ICER directly and additional calculation outside the model has to be undertaken, or authors may prefer not to recommend a particular product.

With the exception of the HPV studies, limited effort was made to compare non-vaccine comparator interventions; this is a particularly egregious omission in the case of rotavirus where the literature suggests highly cost-effective non-vaccine interventions such as breastfeeding promotion. [10] However, HPV vaccine evaluations are including non-vaccine interventions such as screening and treatment. It is hoped that the new UNIVAC model will improve on this issue.

Figure A6. Percentage of studies adhering to good practices for reporting health economic evaluations

The ProVac studies rarely define or discuss the thresholds used to assess cost-effectiveness, which is a limitation given that the program is intended to inform a yes-or-no decision on vaccine adoption. The threshold is clearly stated in the model itself, but this had not made its way systematically into country study write-ups.

In non-ProVac evaluations, mostly funded by manufacturers, it is unsurprising that the pertinence of the threshold is not discussed given that it is in the manufacturers’ interest to justify their product’s cost-effectiveness and a GDP-based threshold is commonly in use. However, a more critical view of the thresholds may be an opportunity to differentiate ProVac evaluations, given that opportunity costs in low-resource health systems are likely not represented by a GDP per capita threshold. Indeed, recent research on even the UK system suggests that a GDP per capita threshold is much higher than the actual opportunity costs of introducing a new technology, [11] and it seems reasonable to assume that trade-offs are more significant in low-spend Latin American health systems.

*Quality of Evidence*

Figure A7 illustrates large variability in the type of baseline clinical data used in studies. Both ProVac and non-ProVac studies use the same types of evidence to estimate baseline clinical values.

Figure A7. Baseline clinical data

For the measurement of the clinical effect used in the studies, the absence of meta-analyses and clinical trials in the international literature is a serious limitation for the quality of the information available to feed the model.

In the case of pneumococcal vaccine, in the absence of clinical trials that permit the determination of the magnitude of the effect of PCV10 and PCV13, study authors have had to extrapolate the clinical effects based on different products for which information is available (PCV7 and PCV 11), making an adjustment for the serotype coverage and the prevalence of the circulating serotypes. This extrapolation is based on limited empirical evidence. This issue can also be addressed with the kinds of calibrated or validated models found among the non-ProVac studies for other diseases, as is the case in the HPV models.

In comparing the quality of evidence on the magnitude of effects for PCV 10 and 13 (excluding PCV 7 for which there are only non-ProVac evaluations), ProVac evaluations are higher quality; although they lack RCT information on the specific products, they do explicitly state the way in which extrapolations of effect sizes were undertaken (see figure A8).

With the exception of the HPV studies, limited effort was made to compare non-vaccine comparator interventions; this is a particularly egregious omission in the case of rotavirus where the literature suggests highly cost-effective non-vaccine interventions such as breastfeeding promotion. [10] However, HPV vaccine evaluations are including non-vaccine interventions such as screening and treatment. It is hoped that the new UNIVAC model will improve on this issue.

Figure A8: Clinical effect sizes in pneumococcal (for comparability purposes just PCV 10 and PCV13, without PCV7)

With respect to the clinical effect of rotavirus vaccines, 7 non-ProVac studies used clinical trials to establish effect sizes for RV1 [12] and RV5 [13]. In the case of ProVac studies (n=3), Albania and Guatemala [14] [15] cite other studies’ efficacy estimates that did not use the same vaccine. [16] [17] In both studies, for example, RV1 efficacy estimates are used for RV5 with no discussion of any kind. For this reason, the quality of evidence for effect sizes in these two studies was assessed as “Not clearly stated”. The remaining ProVac study [18] uses clinical trials but also used information on a similar product to estimate the efficacy of the vaccine on non-severe cases of RV1, and was therefore classified as “5 Explicit use of data from similar product without extrapolation”. In figure A9, we compare the quality of information on clinical effects used in ProVac and non-ProVac studies, finding that an important share of both do not clearly explain the sources of data used to estimate the effect size.

Figure A9: Clinical effect sizes in rotavirus

In the case of economic evaluations on variants of the HPV vaccine conducted with ProVac support, the study on Guatemala [20] uses two randomized control trials [21] [22] as the basis for its efficacy estimates of HPV2 and HPV4 vaccines respectively. For this reason, this study was classified as using “3+ Meta-analysis of placebo-controlled RCTs with similar trial populations, measuring the surrogate outcomes”. Other ProVac studies use the results of two publications to assess efficacy [22] [23]. Lu et al (2011) is a meta-analysis of seven clinical trial and Muñoz is a randomized clinical trial. However, it is possible to have a discussion of the applicability of these studies –which in some cases focus on different products (vaccine against serotypes 6 and 11)- to the evaluated vaccines or to different age groups. For example, in the case of Jamaica [24], the Munoz et al study is used, yet this study is not related to the evaluated bivalent vaccine, but instead to the tetravalent vaccine which may be more effective if more included serotypes are indeed more effective against disease.^[[5]](#footnote-5)^ [21] In spite of this difference, the estimates of efficacy are used with no analysis or discussion. The Lu et al study is a meta-analysis that pools efficacy estimates of both bivalent and tetravalent HPV vaccines yet these pooled average results are used without mention of this fact. Further, the Munoz et al study is carried out in a population of women aged 24-25 years old yet these efficacy estimates are extrapolated to other age groups with no mention or analysis. [both of these issues have been corrected in the published versions of the country studies in Vaccine] Non-ProVac HPV studies use models that include calibration or validation, where actual cases of cancer observed in a population are used to adjust the model-generated number of cases.^[[6]](#footnote-6)^ While cancer and tumor registries are generally incomplete or poor quality in many countries of the region, they are improving over time, and it is also possible to use cases from similar populations in other countries.

It is important to note that the currently-available clinical trials on HPV vaccine do not include cancer outcomes or DALYs. The outcomes of these studies only analyze non-neoplasic lesions. Therefore, a series of assumptions are used to estimate the disability or premature death that may be related to the presence of non-neoplasic lesions. In ProVac studies, there is no discussion of the assumptions necessary to associate the efficacy of the vaccines on non-neoplasic lesions (that do not cause disability or death) with an impact on DALYs; ie, there is no direct discussion or empirical analysis of the probability that a non-cancerous lesion will convert into cancer in a given time period. In non-ProVac HPV studies, several studies make their assumptions on the connection between non-neoplasic lesions and DALY impact explicit.

Figure A10. Clinical effect sizes HPV vaccines (without screening)

In terms of evidence on costs, ProVac studies are more likely to use better sources of data on costs. In general, ProVac studies have made a significant effort to compile up-to-date, representative and reliable data on frequencies of utilization and unit costs. However, almost 40% of cost data used comes from data sources that are not described in the report, and are thus classified as unknown. This does not necessarily mean that the information is actually unknown but that study reports do not provide detail about data sources.

Figure A11. Quality of evidence on costs

**References**

[1] Drummond M, et al. Key principles for the improved conduct of health technology assessments for resource allocation decisions. International journal of technology assessment in health care 2008;24.3:244-258.

[2] Roberts M, et al. Conceptualizing a Model A Report of the ISPOR-SMDM Modeling Good Research Practices Task Force–2. Medical Decision Making 2012;32.5:678-689.

[3] Langer 2012

[4] Eddy, DM, W Hollingworth, JJ Caro, J Tsevat, KM McDonald, JB Wong, and ISPOR-SMDM Modeling Good Research Practices Task Force. Model transparency and validation: A report of the ISPOR–SMDM Modeling Good Research Practices Task Force-7. Value in Health 2012;15.6:843–850.

[5] Halpern, EF, Weinstein, MC, Hunink, MG, et al. Representing both first- and second-order uncertainties by Monte Carlo simulation for groups of patients. Med Decis Making. 2000;20:314–322.

[6] Instituto Nacional de Salud. Estudio de costo efectividad de las vacunas deca- y trece-valente para la prevención de enfermedad asociada a Streptococcus pneumoniae en niños menores de 5 años en Perú. Ministerio de Salud Peru 2014.

[7] Costa Rica: Estudio costo efectividad de la vacuna conjugada contra el neumococo en Costa Rica. Ministerio de Salud Costa Rica 2012.

[8] Aldunate, E. Evaluación de Programas y Proyectos. Available from: http://www.cepal.org/ilpes/noticias/paginas/0/20270/eval_proy_ealdunate.ppt [Accessed October 23, 2015].

[9] Urueña A, Analía, et al. Cost-effectiveness analysis of the 10-and 13-valent pneumococcal conjugate vaccines in Argentina. Vaccine 2011; 29.31: 4963-4972.

[10] Rheingans R, et al. Potential cost-effectiveness of vaccination for rotavirus gastroenteritis in eight Latin American and Caribbean countries. Revista Panamericana de Salud Pública 2007;21.4:205-216.

[11] University of York – Center for Health Economics. Methods for estimation of the NICE cost-effectiveness threshold. Available from: https://www.york.ac.uk/che/research/teehta/thresholds/. [Accessed October 22, 2015].

[12] Soárez P, et al. Cost-effectiveness analysis of routine rotavirus vaccination in Brazil. Revista Panamericana de Salud Pública 2008;23.4:221-230.

[13] Valencia-Mendoza A, et al. Cost-effectiveness of introducing a rotavirus vaccine in developing countries: the case of Mexico. BMC Infectious Diseases 2008;8.1:103.

[14] Preza et al 2013

[15] Ministerio de Salud Pública y Asistencia Social 2012

[16] Linhares A, et al. Efficacy and safety of an oral live attenuated human rotavirus vaccine against rotavirus gastroenteritis during the first 2 years of life in Latin American infants: a randomised, double-blind, placebo-controlled phase III study. The Lancet 2008; 371.9619: 1181-1189.

[17] Ruiz-Palacios G, et al. Safety and efficacy of an attenuated vaccine against severe rotavirus gastroenteritis. New England Journal of Medicine 2006; 354.1: 11-22.

[18] Urueña A, et al. Cost-effectiveness analysis of rotavirus vaccination in Argentina. Vaccine 2015; 33 : A126-A134.

[19] Patel M, et al. "Removing the age restrictions for rotavirus vaccination: a benefit-risk modeling analysis. 2012: e1001330.

[20] (Ministerio de Salud Pública y Asistencia social & Organización Panamericana de la Salud, 2013)

[21] Lehtinen M, et al. Overall efficacy of HPV-16/18 AS04-adjuvanted vaccine against grade 3 or greater cervical intraepithelial neoplasia: 4-year end-of-study analysis of the randomised, double-blind PATRICIA trial. The lancet oncology 2012;13.1 : 89-99.

[22] Muñoz N, et al. Safety, immunogenicity, and efficacy of quadrivalent human papillomavirus (types 6, 11, 16, 18) recombinant vaccine in women aged 24–45 years: a randomised, double-blind trial. The Lancet 2009;373.9679:1949-1957.

[23] Lu B, et al. Efficacy and safety of prophylactic vaccines against cervical HPV infection and diseases among women: a systematic review & meta-analysis. BMC infectious diseases 2011;11.1: 13.

[24] (St Croix, 2012)

[25] Reynales-Shigematsu L, Rodrigues E, and Lazcano-Ponce E. Cost-effectiveness analysis of a quadrivalent human papilloma virus vaccine in Mexico. Archives of medical research 2009;40.6: 503-513.

1. ProVac program staff notes: “ProVac models do a lot more than most other models in this regard! There is a dedicated web site for evidence review which is pretty unique. A quality grading is difficult to assign to international agency parameters (e.g. UNPOP demography, World Bank GDP per capita etc), hence they are left blank deliberately and only research study evidence is quality graded.” [↑](#footnote-ref-1)
2. Pneumonia; Bacterial Pneumonia; Pneumococcal Infections; Pneumococcal Vaccines; Rotavirus; Rotavirus vaccines; Rotavirus infections; Diarrhea; Infantile Diarrhea; Influenza; Influenza Vaccines; Papillomavirus Vaccines; Cervical Neoplasms; Uterine Cervical Dysplasia; Cervical Intraepithelial Neoplasia; Economic evaluation; Medical economics; Pharmacoeconomics; Cost-effectiveness; Cost-utility; Cost-minimization; Cost-minimisation; Cost-benefit analysis [↑](#footnote-ref-2)
3. The Guatemala pneumoccocal vaccine study seems to contain an error, transposing the PCV10 with the PCV13 cost-effectiveness ratios, as described below. [↑](#footnote-ref-3)
4. ProVac program staff notes: “This depends on policy question of course. If vaccine prices is not substantially different and modeled effectiveness differences minimum, then a relevant policy question may be: is the introduction of an HPV vaccine cost-effective?” [↑](#footnote-ref-4)
5. According to information provided by the ProVac team, this is an error in the report and it was not actually used in the model runs. [↑](#footnote-ref-5)
6. A good example of a calibrated model can be found in [25]. [↑](#footnote-ref-6)
